# Does a Dedicated Unit for the Treatment of Hip Fractures Improve Acute Outcomes?

**DOI:** 10.1155/2014/385701

**Published:** 2014-11-20

**Authors:** Al-achraf Khoriati, Wael Dandachli, Rupinderbir Deol, Nicholas de Roeck

**Affiliations:** The Orthopaedic Department, Lister Hospital, Stevenage SG1 4AB, UK

## Abstract

The aim of this study is to establish whether management of patients in a unit dedicated to the treatment of hip fractures improves acute outcomes. We prospectively studied 300 patients with hip fractures in two separate groups. Patients in Group 1 were operated on in a mixed trauma unit and recovered in a traditional trauma ward. Patients in Group 2 were operated on in dedicated theatres and recovered in a unit which catered exclusively for hip fractures. The ages, ASA grades, and type of procedure performed in the two groups were comparable. The 30-day mortality rate in Group 2 was 9% as opposed to 12% in Group 1 (*P* = 0.34). The inpatient length of stay was significantly lower in Group 2 (18 days versus 25 days; *P* = 0.0002) and so was the time taken to operate (28 hours versus 34 hours; *P* = 0.04). A greater percentage of patients in Group 2 were discharged home as opposed to a nursing home (75% versus 67%). This difference approached significance (*P* = 0.18). We conclude that prioritisation and prompt management of patients with hip fractures in a dedicated unit significantly improve time to surgery and significantly decrease length of stay.

## 1. Introduction

Hip fractures are an increasing problem in the United Kingdom. They encompass all fractures that occur between the edge of the femoral head and 5 centimetres below the lesser trochanter [1]. The average annual incidence of hip fractures is on the increase and currently lies at between 70,000 and 75,000 [1] in the UK. These fractures are associated with a high level of morbidity and mortality with approximately 10% of patients dying within one month of their hospital admission and around one-third dying within a year [1]. The financial implications of managing patients with hip fractures are also significant, with the annual cost (including medical and social care) rising to *£*2 billion per annum [1] in the United Kingdom.

The high mortality rate and cost of managing hip fractures have led to the development of a number of guidelines which clearly set out the optimum manner in which patients with hip fractures should be managed. These include the BOAST1 (British Orthopaedic Association Standards of Treatment) [2] as well as the NICE (National Institute for Clinical Excellence) guidelines. The National Hip Fracture Database [3] (NHFD) has also been established in order to study trends and outcomes in the treatment of these fractures. Health care trusts (bodies responsible for leading and coordinating health care at a local level) can receive financial benefit by means of Best Practice Tariff (BPT) if data is entered onto the NHFD demonstrating that certain standards have been adhered to.

In East and North Hertfordshire National Health Service (NHS) trust, the management of patients with hip fractures has come under both scrutiny and reform. The trust admits approximately 480 patients per year with this diagnosis. Previously, patients were treated both at the Lister Hospital in Stevenage and at the Queen Elizabeth II (QE2) Hospital in Welwyn Garden City. A traditional model of care was in place with patients under the care of orthopaedic surgeons only in a trauma ward. Patients often had delays to surgery owing to inadequate theatre capacity or underwent surgery out of hours performed by more junior surgeons. There was no regular preoperative orthogeriatric provision provided.

There was therefore concern within the trust about the standards of care patients with hip fractures were receiving, as comparative data from sources such as Dr. Foster [4] demonstrated poor performance, particularly in hospital standardised mortality and length of stay. In October 2011 the trust underwent a major reconfiguration of services as part of the process towards merging into one acute site in 2014. The orthopaedic department used the opportunity created by these changes to create a specialist hip fracture unit.

This unit was comprised of theatres with staff and equipment dedicated to the treatment of hip fractures. The staff manning the theatres was specifically trained on the use of equipment relating to hip fracture surgery. An orthogeriatric consultant, registrar, and two senior house officers would also be assigned specifically to manage these patients' medical needs (often complex). A hip fracture nurse coordinator would also be employed to manage the complex social and rehabilitation issues surrounding these patients' discharges.

**Figure 1:**
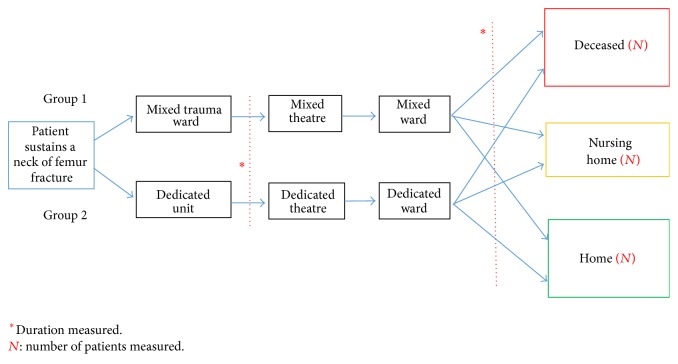
A flow chart describing the step by step management of each patient group as well as the parameters measured.

The aim of this study is to investigate whether or not this dedicated unit improved hospital outcomes acutely. These outcomes were time to operate, length of stay, 30-day mortality, and the percentage of patients discharged to their own homes. A flow chart illustrating the management pathway of each group is included (Figure 1). For the purpose of clarity we will refer to them as Group 1 (the patients treated in a mixed unit) and Group 2 (the patients treated in the dedicated unit).

## 2. Patients and Methods

Data on a total of 300 patients was prospectively collected. One hundred and fifty patients were studied from both the Lister Hospital and Welwyn Garden City and were treated between November 2010 and March 2011. These constituted Group 1. The second group of patients was studied in a similar manner at the new hip fracture unit (Group 2). These patients were consecutively treated between April 2012 and August 2012.

The unit itself was opened in October 2011. The time periods were selected to allow for the unit to overcome any initial logistical problems and to get up and running properly.

Patients' notes were reviewed both on admission and on discharge. Age, gender, time to operation (from admission to the emergency department), type of operation, ASA grade, and length of hospital stay were all recorded. The number of patients returning to their own homes (as opposed to a nursing home) after discharge was also noted. Patients with periprosthetic and pathological fractures were excluded because such patients often require additional investigations and equipment to be ordered before surgery. This in turn would alter both the time to operation and the length of stay. The mortality rate in such a group of patients would inevitably be higher.

The 30-day mortality was calculated as a percentage of the total number of patients treated.

### 2.1. Statistical Analysis

Using SPSS 14.0 statistical software (SPSS Inc., Chicago, USA), the data was tested for normality using the Kolmogorov-Smirnov test. Both the data for length of stay and time to operation were normally distributed. The* t*-test was therefore used to assess any statistically significant difference between the two groups. The significance level was chosen at 0.05. The two-tailed* Z*-test for comparing two population proportions was used for comparing the data for 30-day mortality. This was also used for the data on patients discharged home.

## 3. Results

The patients in the two groups had comparable ASA grades. These are displayed in Table 1. The type of operation for each group is listed in Table 2. The distribution of operation types was similar in each group. The mean age in Group 1 was 82.4 years and the male : female ratio was 1 : 3.8. The mean in Group 2 was 81.7 years and the male : female ratio was 1 : 3.1.

The 30-day mortality rate for Group 1 was 12% as opposed to 9% in Group 2. The difference was not statistically significant (*P* = 0.34).

The average length of stay for patients in Group 1 was 25 days, whereas in Group 2 it was 18 days. The difference was statistically significant (*P* = 0.0002).

The mean time taken from admission to the emergency department to the operation was 34 hours in Group 1, whereas in Group 2 it was 28 hours; this was significantly lower than the former group (*P* = 0.04).

Additionally, in Group 2, all operations were performed in daytime hours on a list exclusively for patients with hip fractures, with staff trained specifically for such procedures, and in the presence of a consultant orthopaedic surgeon. This is in direct contrast with the patients from Group 1, which were treated either on a half day trauma list (69%) or on the surgical emergency list (31%). The surgical emergency list ran continuously from noon to midnight and was staffed by personnel who were not exclusively trained in orthopaedic procedures.

The number of patients admitted from their own home in Group 1 was 123. Of these patients, 83 (67%) were discharged back to their homes. 114 Patients were admitted from home in Group 2; 86 of them (75%) returned there. The difference approached significance (*P* = 0.18).

## 4. Discussion

The management of patients with hip fractures involves a complex cascade of events, the ultimate goal of which is to reduce both morbidity and mortality in individuals who have sustained a life-threatening injury. A number of guidelines have been adopted nationwide in order to improve the management of such patients. Among these are the BOAST [2] guidelines which state the following.

- Patients with hip fractures should be managed by a multidisciplinary team (surgeons/orthogeriatricians/anaesthetists/specialist nurses).
- Specialists need to see the patients and initiate their management rapidly.
- Correctable comorbidities must be identified and treated immediately.
- Consultants should be present at all operative lists, which should be planned lists.

Our goal was to ascertain whether or not the implementation of these guidelines within our dedicated hip fracture unit would in fact lead to an improvement in several parameters. These included the time taken to operate, the mortality at thirty days, hospital length of stay, and whether or not a patient was able to return home.

It has been well established that both rapid diagnosis and intervention carry a profound effect on the outcomes of patients with hip fractures [1]. It has been shown that the treatment of patients with hip fractures on dedicated trauma lists reduces their postoperative complications [5] and morbidity and mortality [6]. Operating in daylight hours decreases morbidity in these patients [7, 8]. This is thought to be down to a number of factors including surgeon's fatigue, decreased medical and critical care, and specialist theatre staff availability (the available staff may not be familiar with the required equipment) [9–11].

Operative delays are also a major issue in the management of patients with hip fractures. The risk of a patient dying following a hip fracture has been found to increase following a delay of more than 48 hours [12, 13]. Zuckerman et al. reported that this overall risk is in fact doubled at one year postoperatively [14]. Several existing guidelines (BOAST, NICE, and National Service Framework for Older People) advise rapid review by an orthogeriatric team in order to swiftly optimise patients for surgical intervention. It has been shown that the implementation of this process results in decreased mortality as well as a reduced length of hospital stay [15]. It is therefore important to reconcile the need for rapid surgical intervention with the need for preoperative orthogeriatric assessment.

The creation of our hip fracture unit revolved around factoring in all of the above. Patients from Group 2 were all triaged directly and rapidly from the emergency department and were seen by a surgical trainee within a maximum of four hours from their admission to hospital. They were then assessed by an anaesthetist and an orthogeriatrician within 12 hours of admission in order to optimise their progress to theatres. Postoperatively, all care and rehabilitation is carried out by physiotherapists and nurses accustomed to the management of patients with hip fractures. These would then help the patients mobilise and rehabilitate faster leading to a more rapid discharge.

The Best Practice Tariff (BPT) for hip fractures came into effect in April 2010 as part of the recommendations related to Lord Darzi's NHS Next Stage Review report [16]. It set out a number of standards that needed to be met by various health care trusts. For each patient that is successfully treated by the standards set out in the BPT, a trust receives a substantial financial sum. Thus, an incentive based system exists for the treatment of patients with fractured hips.

In order for the trust to meet the requirements defined by BPT, all patients with fractured hips must

- undergo surgery 36 hours from arrival in the emergency department,
- be admitted under the joint care of a consultant geriatrician and a consultant orthopaedic surgeon,
- be admitted using an assessment protocol agreed on by geriatric medicine, orthopaedic surgery, and anaesthesia,
- be assessed by a geriatrician or specialist trainee in the perioperative period (defined as within 72 hours of admission),
- be managed by a postoperative geriatrician-directed multiprofessional rehabilitation team,
- undergo fracture prevention assessments (falls and bone health).

The BPT in itself was necessary in our trust as a funding measure to secure the services (orthogeriatricians/specialist nurses and equipment) required to open our dedicated unit.

There is scant work in the literature looking into the implementation and overall impact of the suggested national guidelines regarding patients with fractured hips. Studies on the impact of individual components of management such as dedicated operating theatres and time of operation [17] have shown that hip fractures are best managed during daytime hours and by a dedicated team. A study by Mallick et al. [15] considered the effects of organisational changes on fracture neck of femur management. The study looked into the impact of orthogeriatricians, trauma coordinators, clinical aides, and discharge nurses on both the number of patients going to theatre within 48 hours and the in-hospital mortality rates of patients with hip fractures. The impact of the aforementioned changes was found to be positive, with the in-hospital mortality decreasing from 16.5% in 2005/6 to 10.9% in 2007/8 and the number of patients going to theatre within 48 h of admission rising dramatically from 38.5% in 2005/6 to 90% in 2007/8. These findings were supported by Dy et al. [18] who found that multidisciplinary collaboration for patients with hip fractures decreased the likelihood of inpatient complications in male patients.

The importance of our study in relation to the existing material is that it considers all of these factors: dedicated theatres and staff, operating hours, and a multidisciplinary approach. The decrease in the time taken to operate from admission reflects this and is supported by other studies [19]. The availability of theatres dedicated to hip trauma also facilitates rapid intervention and eliminates interference of other cases with the prioritisation of hip operations. We believe that this rapid intervention in turn is responsible for the improved in-hospital mortality as well as the decrease in in-hospital stay. We feel that the fact that these cases were performed purely on an orthopaedic list (and not the emergency list) is relevant. The staff is purely trained to work with patients with hip fractures. Additionally, the presence of physiotherapists and nurses that cater exclusively for these patients also facilitates the rapid rehabilitation and appears to have a positive impact on the speed with which patients are able to mobilise and go home (length of stay decreased from an average of 25 days to 18 days).

It is noteworthy that in both groups of patients a similar distribution of cases and ASA mortality grades existed, thus minimising the effects of bias on our study.

This study does however have several limitations. Only 30-day mortality was recorded and therefore it was not possible to establish whether treatment of patients with hip fractures in a dedicated unit has any bearing on long-term mortality. Moreover, mortality may be a crude measure of outcome, and our study did not look into any differences in morbidity or walking ability of the patients. Finally, the fact that we have looked into several parameters means that it is impossible to assign any improvement in outcome to one specific factor. What is demonstrated is purely a combined effect of all our interventions.

This study demonstrates that, by creating a dedicated unit using the BOAST1 and NICE guidance and aiming to deliver care to achieve BPT as defined by the National Hip Fracture Database group, it is possible to reduce length of stay and time to operation. This may also reduce mortality and increase the likelihood of the patient returning to their own home.

In conclusion, patients with hip fractures may be more effectively managed in a dedicated unit. Such cases may then be prioritised and rapidly treated according to national guidelines, which in turn optimises patient care and improves their outcomes after what is essentially a life-threatening injury.

## Figures and Tables

**Table 1:** ASA grades as distributed by group.

	Group 1	Group 2
ASA—1	4	6
ASA—2	49	36
ASA—3	85	97
ASA—4	12	9
ASA—5	0	2

**Table 2:** Operations performed as distributed by group.

	Group 1	Group 2
Cannulated screws/DHS	67	60
IM Nail	20	9
Hemiarthroplasty	59	68
THR	0	9
Nil	4	4

## References

[1] The NICE guidelines regarding treatment of hip fractures. http://www.nice.org.uk/guidance/CG124.

[2] British Orthopaedic Association Standards of Treatment (BOAST) for Hip Fractures https://www.boa.ac.uk/wp-content/uploads/2014/05/BOAST-1-Version-2-Patients-sustaining-a-Fragility-Hip-Fracture.pdf.

[3] The National Hip Fracture Database, 2012 report. http://www.nhfd.co.uk/003/hipfractureR.nsf/0/da44e3a946a14e4180257a6f001eb4db/$FILE/NHFD%20National%20Report%202012.pdf.

[4] Dr Foster UK Medical, Health, and Hospital Guides and Information. http://www.drfosterhealth.co.uk/.

[5] Lemos D., Nilssen E., Khatiwada B. (2009). Dedicated orthopedic trauma theatres: effect on morbidity and mortality in a single trauma centre. *Canadian Journal of Surgery*.

[6] Elder G. M., Harvey E. J., Vaidya R., Guy P., Meek R. N., Aebi M. (2005). The effectiveness of orthopaedic trauma theatres in decreasing morbidity and mortality: a study of 701 displaced subcapital hip fractures in two trauma centres. *Injury*.

[7] Ricci W., Schwappach J., Leighton R., Tornetta P. Is “after hours” surgery associated with adverse outcomes?.

[8] Rogers F. B., Shackford S. R., Keller M. S. (1995). Early fixation reduces morbidity and mortality in elderly patients with hip fractures from low-impact falls. *Journal of Trauma: Injury Infection & Critical Care*.

[9] Kesmezacar H., Ayhan E., Unlu M. C., Seker A., Karaca S. (2010). Predictors of mortality in elderly patients with an intertrochanteric or a femoral neck fracture. *The Journal of Trauma*.

[10] Taffinder N. J., McManus I. C., Gul Y., Russell R. C. G., Darzi A. (1998). Effect of sleep deprivation on surgeons' dexterity on laparoscopy simulator. *The Lancet*.

[11] Gawande A. A., Zinner M. J., Studdert D. M., Brennan T. A. (2003). Analysis of errors reported by surgeons at three teaching hospitals. *Surgery*.

[12] Novack V., Jotkowitz A., Etzion O., Porath A. (2007). Does delay in surgery after hip fracture lead to worse outcomes? A multicenter survey. *International Journal for Quality in Health Care*.

[13] Shiga T., Wajima Z., Ohe Y. (2008). Is operative delay associated with increased mortality of hip fracture patients? Systematic review, meta-analysis, and meta-regression. *Canadian Journal of Anesthesia*.

[14] Zuckerman J. D., Skovron M. L., Koval K. J., Aharonoff G., Frankel V. H. (1995). Postoperative complications and mortality associated with operative delay in older patients who have a fracture of the hip. *Journal of Bone and Joint Surgery—Series A*.

[15] Mallick E., Gulihar A., Taylor G., Furlong A., Pandey R. (2010). Impact of organisational changes on fracture neck of femur management. *Annals of The Royal College of Surgeons of England*.

[16] https://www.gov.uk/government/uploads/system/uploads/attachment_data/file/228836/7432.pdf.

[17] Chacko A. T., Ramirez M. A., Ramappa A. J., Richardson L. C., Appleton P. T., Rodriguez E. K. (2011). Does late night hip surgery affect outcome?. *Journal of Trauma-Injury Infection & Critical Care*.

[18] Dy C. J., Dossous P.-M., Ton Q. V., Hollenberg J. P., Lorich D. G., Lane J. M. (2011). Does a multidisciplinary team decrease complications in male patients with hip fractures?. *Clinical Orthopaedics and Related Research*.

[19] Belmont P. J., Garcia E. J., Romano D., Bader J. O., Nelson K. J., Schoenfeld A. J. (2014). Risk factors for complications and in-hospital mortality following hip fractures: a study using the National Trauma Data Bank. *Archives of Orthopaedic and Trauma Surgery*.

